# Retraction notice for: “Ginsenoside Rd inhibits IL-1β-induced inflammation and degradation of intervertebral disc chondrocytes by increasing IL1RAP ubiquitination” [Braz J Med Biol Res (2019) 52(9): e8525]

**DOI:** 10.1590/1414-431X20198525retraction

**Published:** 2019-12-05

Wei-Jia Zhang https://orcid.org/0000-0002-6427-0056 ^1^, Ying Liu https://orcid.org/0000-0002-6731-1351 ^1^, Jie-Shu Wei https://orcid.org/0000-0001-9178-2631 ^1^, Ya-Li Wu https://orcid.org/0000-0002-6114-5612 ^2^

^1^School of Pharmacy, Xinhua College of Sun Yat-Sen University, Tianhe District, Guangzhou City, Guangzhou, China

^2^School of Rehabilitation Medicine, Xinhua College of Sun Yat-sen University, Tianhe District, Guangzhou City, Guangzhou, China

**Retraction for:** Braz J Med Biol Res | doi: http://10.1590/1414-431x20198525 | PMCID: PMC6694592 | PMID: 31411316

The authors would like to retract the article “Ginsenoside Rd inhibits IL-1β-induced inflammation and degradation of intervertebral disc chondrocytes by increasing IL1RAP ubiquitination” that was published in volume 52 no. 9 (2019) (Epub Aug 12, 2019) in the Brazilian Journal of Medical and Biological Research <http://dx.doi.org/10.1590/1414-431x20198525> PMCID: PMC6694592 | PMID: 31411316.

The Corresponding author Ya-Li Wu states that “there is a conflict between authors concerning the publication of Figure 1 Aa and Ab, which had already been published in a Chinese Journal”. Therefore, this article is being retracted and all authors will be prohibited to publish in the Brazilian Journal of Medical and Biological Research in the future.

